# Patient and public involvement in mobile health-based research for hay fever: a qualitative study of patient and public involvement implementation process

**DOI:** 10.1186/s40900-022-00382-6

**Published:** 2022-09-02

**Authors:** Kenta Fujio, Takenori Inomata, Kumiko Fujisawa, Jaemyoung Sung, Masahiro Nakamura, Masao Iwagami, Kaori Muto, Nobuyuki Ebihara, Masahiro Nakamura, Mitsuhiro Okano, Yasutsugu Akasaki, Yuichi Okumura, Takuma Ide, Shuko Nojiri, Masashi Nagao, Keiichi Fujimoto, Kunihiko Hirosawa, Akira Murakami

**Affiliations:** 1 Department of Ophthalmology, Juntendo University Graduate School of Medicine, 2-1-1 Hongo, Tokyo, 113-0033 Japan; 2 Department of Digital Medicine, Juntendo University Graduate School of Medicine, Tokyo, Japan; 3 Department of Hospital Administration, Juntendo University Graduate School of Medicine, Tokyo, Japan; 4 Department of Public Policy, The Institute of Medical Science, The University of Tokyo, Tokyo, Japan; 5 Precision Health, Department of Bioengineering, Graduate School of Engineering, The University of Tokyo, Tokyo, Japan; 6 Department of Health Services Research, Faculty of Medicine, University of Tsukuba, Tsukuba, Ibaraki Japan; 7 Department of Ophthalmology, Urayasu Hospital, Juntendo University, Chiba, Japan; 8 Department of Otorhinolaryngology, Head and Neck Surgery, Juntendo University Faculty of Medicine, Tokyo, Japan; 9 Department of Otorhinolaryngology, International University of Health and Welfare, Narita, Japan; 10 Medical Technology Innovation Center, Juntendo University, Tokyo, Japan; 11 Department of Orthopedic Surgery, Juntendo University Graduate School of Medicine, Tokyo, Japan; 12 School of Health and Sports Science, Juntendo University, Chiba, Japan

**Keywords:** Patient and public involvement, Public engagement, Patient and public engagement, Mobile health, ResearchKit, Hay fever, Smartphone application, Allergic rhinitis, Allergic conjunctivitis, Pollinosis

## Abstract

**Background:**

Smartphones are being increasingly used for research owing to their multifunctionality and flexibility, and crowdsourced research using smartphone applications (apps) is effective in the early detection and management of chronic diseases. We developed the AllerSearch app to gather real-world data on individual subjective symptoms and lifestyle factors related to hay fever. This study established a foundation for interactive research by adopting novel, diverse perspectives accrued through implementing the principles of patient and public involvement (PPI) in the development of our app.

**Methods:**

Patients and members of the public with a history or family history of hay fever were recruited from November 2019 to December 2021 through a dedicated website, social networking services, and web briefing according to the PPI Guidebook 2019 by the Japan Agency for Medical Research and Development. Nine opinion exchange meetings were held from February 2020 to December 2021 to collect opinions and suggestions for updating the app. After each meeting, interactive evaluations from PPI contributors and researchers were collected. The compiled suggestions were then incorporated into the app, establishing an active feedback loop fed by the consistently interactive infrastructure.

**Results:**

Four PPI contributors (one man and three women) were recruited, and 93 items were added/changed in the in-app survey questionnaire in accordance with discussions from the exchange meetings. The exchange meetings emphasized an atmosphere and opportunity for participants to speak up, ensuring frequent opportunities for them to contribute to the research. In March 2020, a public website was created to display real-time outcomes of the number of participants and users’ hay-fever-preventative behaviors. In August 2020, a new PPI-implemented AllerSearch app was released.

**Conclusions:**

This study marks the first research on clinical smartphone apps for hay fever in Japan that implements PPI throughout its timeline from research and development to the publication of research results. Taking advantage of the distinct perspectives offered by PPI contributors, a step was taken toward actualizing a foundation for an interactive research environment. These results should promote future PPI research and foster the establishment of a social construct that enables PPI efforts in various fields.

**Supplementary Information:**

The online version contains supplementary material available at 10.1186/s40900-022-00382-6.

## Plain English summary

Patient and public involvement (PPI) plays an important role in promoting effective execution of health science research, as well as in the establishment of a social agreement and infrastructure for the care of various diseases, including cancer, chronic diseases, and allergic illnesses. Hay fever is one of the most common allergic diseases, affecting more than 30 million people in Japan. It is known for its myriad factors and diverse presentations. Previously, we developed a mobile health (mHealth) smartphone application (app) for hay fever—AllerSearch—released in February 2018. This app is capable of collecting relevant digital phenotypes and user-provided information, which are used in providing tailored, evidence-based suggestions. To our knowledge, no other studies have been conducted on the implementation of PPI in mHealth. Since hay fever presents with a wide variety of symptoms and risk factors, PPI principles appear well-suited for eliciting insights from the patient/public population and for incorporating new, expert perspectives into the research process. In this study, we included PPI contributors in the research plan, app development, and evaluation. Most notably, the survey questionnaire and user interface of the app was tailored based on PPI feedback. The updated AllerSearch app was released during this study period. Since hay fever is a widespread and variable illness, the multifaceted input from patients and public experts enabled by PPI implementation holds promise for improving society-wide healthcare and in empowering a culture toward medical involvement.

**Supplementary Information:**

The online version contains supplementary material available at 10.1186/s40900-022-00382-6.

## Background

Hay fever is the most common allergic disease—affecting approximately 30 million people in Japan, with future prevalence projected to increase [1, 2]. This can be inferred from clinical practice, as hay fever is one of the most common reasons for hospital visits, which ultimately increases personal and societal medical costs [3, 4]. In addition to its detrimental effect on quality of life (QoL), its negative impact on work productivity—including the various metrics of presenteeism and absenteeism—leads to economic losses on a global scale [5–7].

Hay fever is a multi-organ disease that leads to a wide variety of symptoms including rhinitis, conjunctivitis, pharyngeal symptoms, pollen dermatitis, and general malaise [8]. This is further complicated by its pathologic and presentation heterogeneity observed through widely varying onset ages, presenting symptoms, symptom severity, and prognosis on a case-by-case basis [9]. Currently, hay fever is well-established as a multifactorial disease influenced by environmental, lifestyle, host factors, including pollen, air pollutants, diet, smoking, exercise, family history, and age [10]. Despite the recognition of such variability in presentation and risk factors, the current standard of care fails to effectively tailor hay fever management based on patient characteristics.

This is possibly owing to a lack of understanding of the dynamics between the disease and factors, as well as minimal input from patients on their own individual factors. As such, an ideal strategy to tackle complex pathologies necessitates a holistic approach which considers motivation, access, ease of participation, and areas of further improvements. These factors beyond the typical realm of medicine become crucial—especially for research that requires both immense and sensitive personal data—to effectively perform comprehensive research, maximize participation, and minimize attrition rate; such a development process can benefit from the novel, multifaceted perspectives offered by PPI [9, 11, 12].

Patient and Public Involvement (PPI) encompasses the involvement of patients and the public in various sectors of research, including initial development, execution, and ongoing changes, working in partnership with primary researchers [13]. The search for unmet medical needs and the incorporation of novel, multifaceted perspectives through PPI implementation enables researchers to take advantage of the unique experiences and expertise offered by participants from beyond-medical sectors [9, 14]. Ongoing research on PPI has been showing promising results and is gaining recognition as an important element in improving healthcare and in successful healthcare research [15]. Therefore, efforts to incorporate elements of PPI in all stages of medical research can be invaluable in promoting the effective execution of research in various fields, as well as the establishment of a social agreement and infrastructure that is compatible with the future vision of medicine.

For this purpose, we developed a mobile health (mHealth) smartphone application (app) on hay fever, AllerSearch, with the proactive implementation of PPI to evaluate the value of external participation and multifaceted perspectives in the research and collection of comprehensive big data [9, 12, 16]. mHealth applications and research enable the collection of individualized, real-world big data by taking advantage of the frequent, longitudinal, remote, and real-time nature of a smartphone-driven biosensor data (digital phenotypes) [16–27], which can then be utilized in personalized medical intervention and management. AllerSearch is similarly capable of collecting relevant digital phenotypes, as well as user-provided information, which are used in providing evidence-based suggestions tailored to the provided data [9, 12, 16]. In addition, by reflecting various opinions from PPI in the smartphone app, continuous improvements in collecting previously neglected factors that reflect symptoms and lifestyle factors contributory to hay fever have been made possible.

This study established a foundation for interactive research by adopting novel, diverse perspectives accrued through implementing the principles of PPI in the development of our app.

## Methods

### Smartphone application AllerSearch for hay fever

**Fig. 1:**
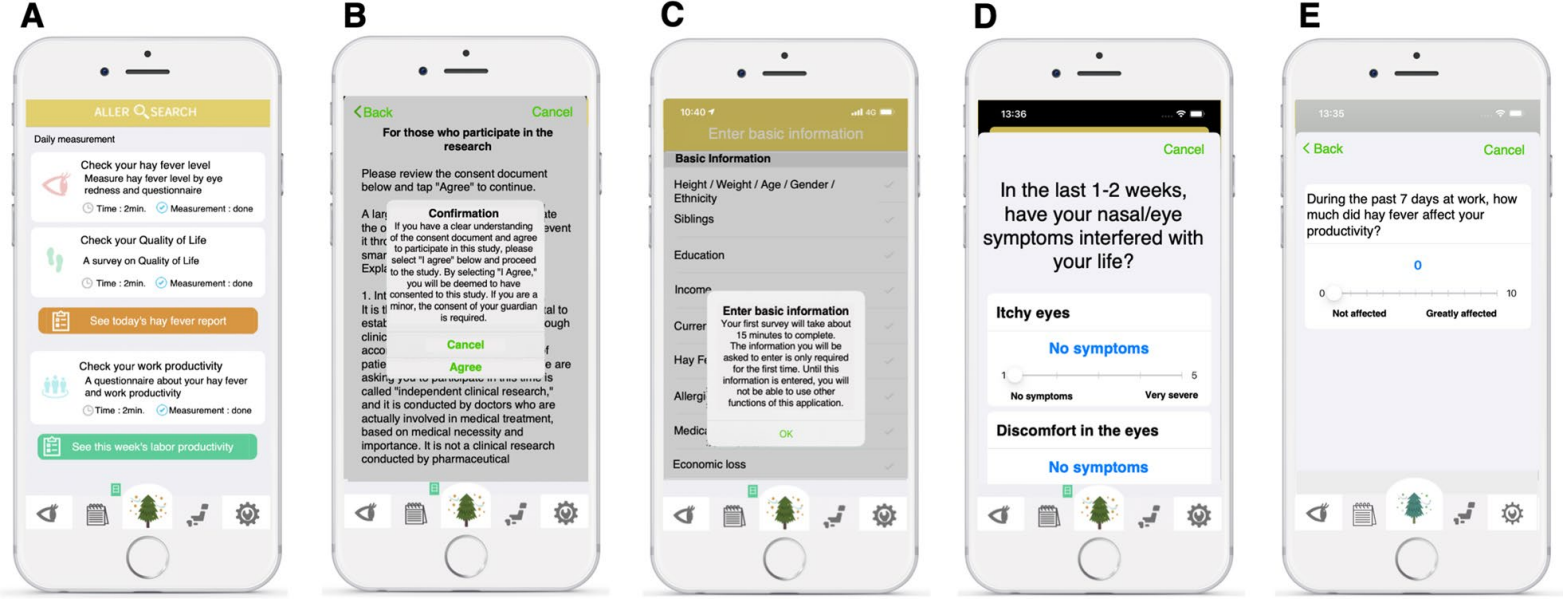
Screenshot of AllerSearch. **A** Top screen of AllerSearch. The app has three daily tasks: checking hay fever level (top), checking quality of life (middle), and checking work productivity (bottom). **B** Screen for electronic informed consent of participants. **C** Screen for entering participant characteristics. **D** Questionnaire for hay fever symptoms. **E** Questionnaires for quality of life related to hay fever

The self-developed iPhone application, AllerSearch, was released as an iOS-based application on ResearchKit in February 2018 [9, 12, 16] under a consignment contract with Juntendo University Graduate School of Medicine and InnoJin, Inc., Tokyo, Japan. The Android version was released on August 26, 2020. AllerSearch aimed at gathering real-world data on individual subjective symptoms and lifestyle factors on hay fever (Fig. 1A). Once the users download the application from the App Store and Google play, an electronic consent form is collected on data usage for research purposes (Fig. 1B). The basic user profile (Fig. 1C) includes: age; biological sex; height; weight; family composition; previous diagnosis of hay fever; medical history of conditions such as hypertension, diabetes, heart disease, respiratory disease, brain disease, liver disease, renal disease, hematologic disease, malignancy, and mental illness; as well as lifestyle and environmental information such as smoking status, exercise pattern, contact lens use, sleep duration, home environment, and preventative behavior regarding hay fever such as use of a mask, eye drops, nasal drops, air purifier, glasses, and eye wash (Additional file 1: Table S1) [16].

Daily hay fever symptoms are evaluated based on a composite score [12, 16], including the nasal symptom score (NSS) and non-nasal symptom score (NNSS) showed in Additional file 1: Table S2. The NSS consists of items pertaining to rhinorrhea, nasal congestion, nasal itching, sneezing, and interference with daily life. The NNSS consists of items pertaining to itchy eyes, watery eyes, eye redness, and itchy ears and mouth. Additionally, AllerSearch collects longitudinal data through patient inputs, smartphone-embedded sensors, and external sources, such as self-preventative measures, conjunctival images, user location, PM2.5 (fine particulate matter), and pollen distribution. The app also requests information on QoL (Additional file 1: Table S3) and work productivity (Additional file 1: Table S4) through the Japanese allergic conjunctival diseases Quality of Life Questionnaire (JACQLQ) [28, 29] and Japanese version of Work Productivity and Activity Impairment: Allergy Specific [30], respectively (Fig. 1D, E). The clinical study was approved by the Juntendo University Hospital, Independent Ethics Committee (Approval Number, 20-243) and adhered to the tenets of the Declaration of Helsinki.

### Recruitment of PPI contributors

The recruitment criteria for PPI contributors for this study was determined based on the PPI Guidebook published by the Japan Agency for Medical Research and Development (AMED) in 2019 [31]. Guidance for Reporting Involvement of Patients and Public [32] and previous reports from foreign countries [33, 34] were utilized as an ancillary tool for fine adjustments in the recruitment guideline, as fluency was only expected in the Japanese language and not English from the PPI contributors. The target criteria for a successful recruitment included: (1) 10 participants with a minimum age of 20 years regardless of sex; (2) adequate participant knowledge on or experience with hay fever, either through personal or family history; and (3) capability to participate in online meetings using an electronic device (Table 1). Additionally, as part of the recruitment process, information regarding the research was provided, including its background, researchers’ request to PPI contributors, meeting details (location, number of participants, transportation, online access availability, etc.), meeting schedules, compensation (transportation fee and 1000 Japanese yen/hour payments), and contact details.

**Table 1:** The requirements of open recruitment

Number of applicants	≤ 10 persons
Age	≥ 20-years-old
PPI implementation period	January 2020–March 2022
Location	Juntendo University Graduate School of Medicine, Tokyo, Japan or Online
Selection criteria	Those who have personal or family history of hay fever Those who can understand the purpose of PPI Those who have the discussion skills for the PPI meetings Those who have the computer skills for online meetings
Application procedure	Document screening

PPI, patient and public involvement

**Fig. 2:**
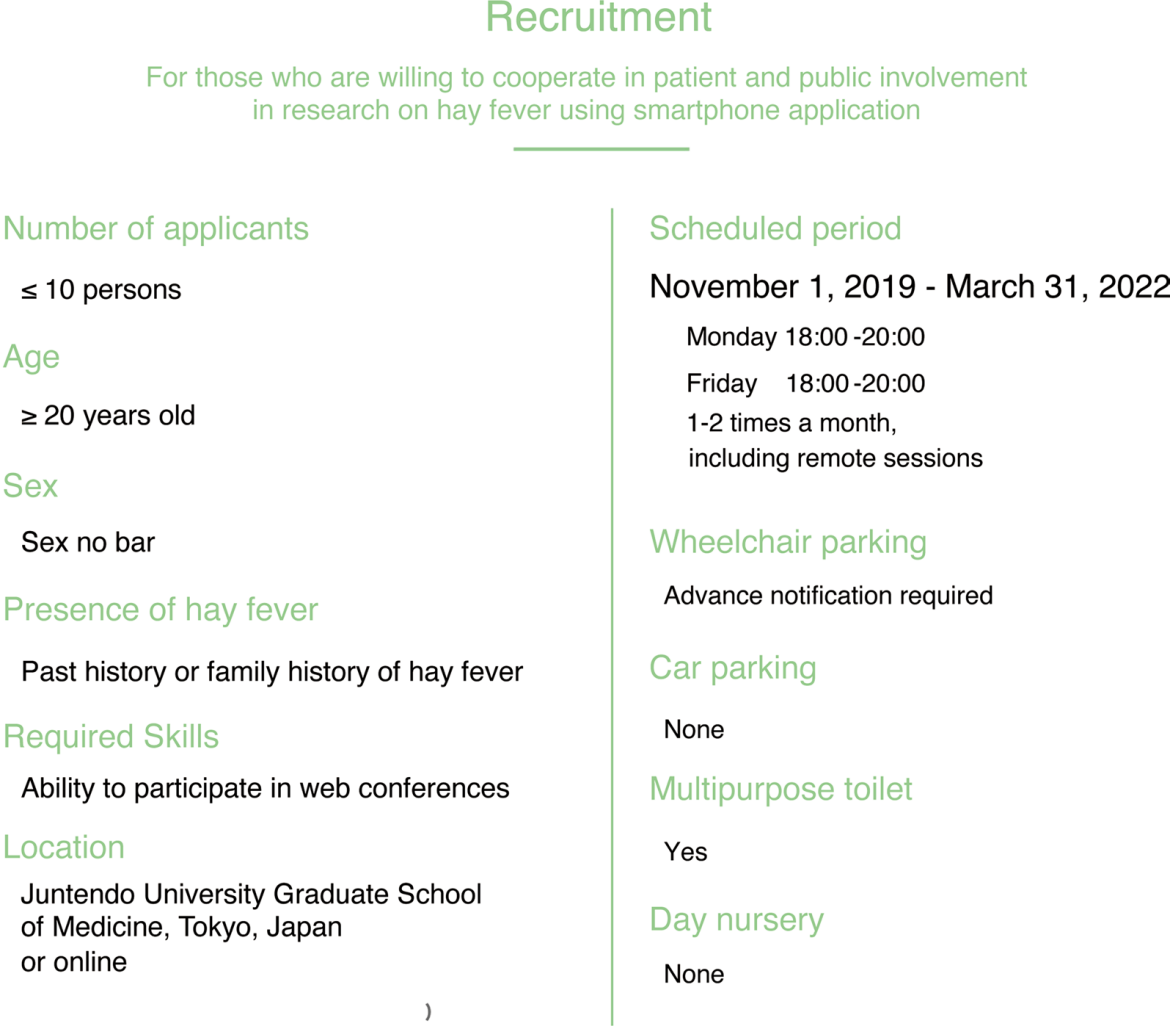
Recruitment page for patient and public involvement contributors on the AllerSearch website. The application guidelines were posted on the AllerSearch website (https://allergy-search.com/). Resumes can be downloaded on this website, and applicants of this study fill out the resume and send it via email

Open recruitment of PPI contributors was conducted from November 2019 to December 2021 through the AllerSearch webpage (Fig. 2) and social networking service (SNS) providers such as Facebook (https://www.facebook.com/allersearchapp/) and Twitter (https://twitter.com/allersearch). To facilitate an inclusive environment and elicit diverse opinions from PPI contributors, the selection committee deliberated on various applicant factors including age, sex, experiential knowledge of hay fever, and the capability to express their opinions objectively. Each participant was subject to an interview process to comprehensively assess the above factors prior to final approval by the selection committee. Ultimately, the selection criteria were formulated according to the applicants’ medical history or family history of hay fever, ability to provide unique, knowledgeable perspectives on the research, as well as on the need to obtain suggestions from various demographics concerning, but not limited to, age, sex, and profession.

### Standard procedure for PPI meetings

**Fig. 3:**
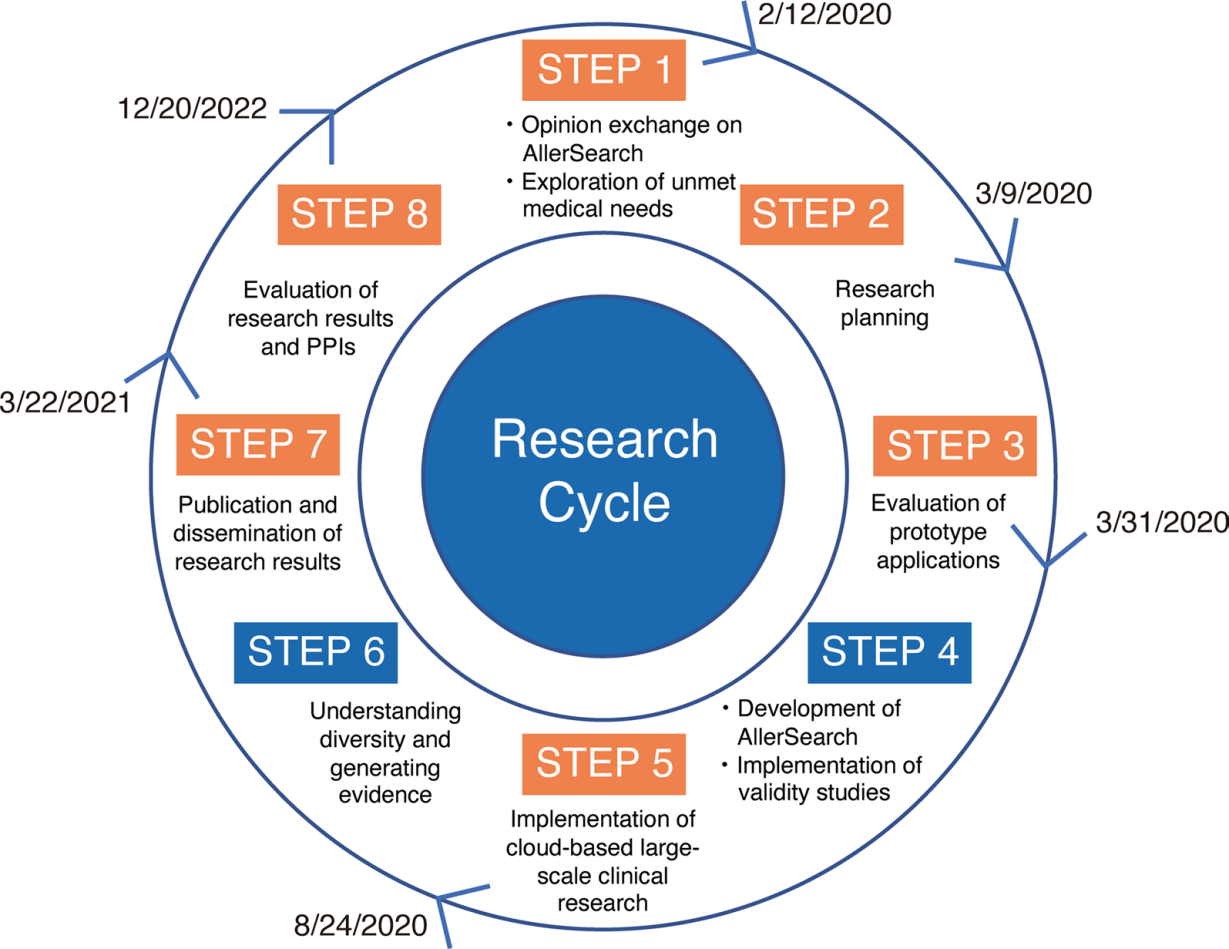
Schematic of the eight steps for PPI implement in this study based on PPI Guidebook. A consistent PPI meeting was held with eight steps reference to PPI Guidebook published by the Japan Agency for Medical Research and Development. The discussion proceeded by identifying which step we are currently following based on these eight steps. STEP 1, 2, 3, 5, 7, and 8 highlighted in orange were done with the PPI committee members, and a timeline is provided for each step. PPI, patient and public involvement

PPI meetings were held in accordance with the eight steps outlined by the PPI Guidebook published by the AMED in 2019 [31] (Fig. 3) to maintain consistency in every meeting. To effectively deliver the details of the research to the PPI contributors, relevant documents were provided prior to each meeting along with an overview and instruction on using the AllerSearch application. Each PPI meeting was facilitated by expert members on PPI (KFujisawa and KM), and their expertise determined by their previous experience on releasing the AMED PPI Guidebook [31]. After each meeting, an interactive evaluation on the meeting from both the PPI contributors and researchers was conducted (Table 2).

**Table 2:** The result of interactive evaluation from both the PPI contributors and researchers on the meeting

PPI contributor, number	1st meeting	2nd meeting	3rd meeting	4th meeting	5th meeting	6th meeting	7th meeting	8th meeting	9th meeting	Total
	n = 3	n = 3	n = 3	n = 2	n = 2	n = 3	n = 3	n = 4	n = 3	N = 26
(1) Difficulty of the materials provided in advance by the research team, 0–4, median (IQR)	4 (3.5–4)	4 (4–4)	4 (4–4)	4 (4–4)	4 (4–4)	4 (4–4)	2 (0–4)	4 (3.75–4)	4 (2–4)	4 (4–4)
(2) Number of materials provided in advance by the research team, 0–4, median (IQR)	4 (3.5–4)	4 (4–4)	4 (4–4)	4 (4–4)	4 (4–4)	4 (4–4)	2 (0–4)	3.5 (2.5–4)	0 (0–2)	4 (4–4)
(3) Timing of materials provided in advance by the research team, 0–4, median (IQR)	4 (3.5–4)	4 (4–4)	4 (4–4)	3.5 (3.25–3.75)	3.5 (3.25–3.75)	3 (3–3.5)	1.5 (0–3.25)	3 (2.25–3.25)	0 (0–2)	4 (3–4)
(4) Frequency of use of technical terms and English in explanations, 0–2, median (IQR)	2 (2–2)	2 (2–2)	2 (2–2)	2 (2–2)	2 (2–2)	2 (2–2)	2 (1.5–2)	1.5 (1–2)	2 (1–2)	2 (2–)
(5) Difficulty of the agenda, 0–3, median (IQR)	3 (3–3)	3 (3–3)	3 (3–3)	3 (3–3)	3 (3–3)	3 (3–3)	3 (3–3)	2.5 (1.5–3)	3 (3–3)	3 (3–3)
(6) Atmosphere for the PPI contributors to speak, 0–2, median (IQR)	2 (2–2)	2 (2–2)	2 (2–2)	2 (2–2)	2 (2–2)	2 (2–2)	2 (2–2)	1 (0–2)	2 (2–2)	2 (2–2)
(7) Clarity of opinions and roles that the research team is seeking from PPI contributors, 0–2, median (IQR)	2 (2–2)	2 (2–2)	2 (2–2)	2 (2–2)	2 (2–2)	2 (2–2)	2 (2–2)	2 (1.5–2)	2 (1–2)	2 (2–2)
(8) Opportunities for PPI contributors to speak, 0–2, median (IQR)	2 (2–2)	2 (1–2)	2 (2–2)	2 (2–2)	2 (2–2)	2 (2–2)	2 (2–2)	2 (2–2)	2 (2–2)	2 (2–2)
(9) Were you able to say what you wanted to say? 0–2, median (IQR)	2 (2–2)	2 (1.5–2)	2 (1.5–2)	1.5 (1.25–1.75)	2 (2–2)	2 (1–2)	2 (2–2)	1 (1–1.25)	2 (2–2)	2 (1.5–2)
(10) Did the PPI contributors fulfilled the role the research team had asked of them? 0–2, median (IQR)	2 (2–2)	0 (0–1)	0 (0–1)	2 (2–2)	2 (2–2)	2 (1–2)	2 (1.5–2)	0 (0–0.5)	2 (2–2)	2 (0–2)

PPI researcher, number	1st meeting	2nd meeting	3rd meeting	4th meeting	5th meeting	6th meeting	7th meeting	8th meeting	9th meeting	Total
	n = 4	n = 8	n = 8	n = 10	n = 8	n = 5	n = 4	n = 7	n = 8	N = 62
(1) Difficulty of the materials provided in advance by the research team, 0–4, median (IQR)	2 (0–4)	4 (4–4)	4 (4–4)	4 (4–4)	4 (4–4)	4 (4–4)	2 (0–4)	4 (4–4)	4 (0–4)	4 (4–4)
(2) Number of materials provided in advance by the research team, 0–4, median (IQR)	2 (0–4)	4 (4–4)	4 (4–4)	4 (4–4)	4 (4–4)	4 (3–4)	2 (0–4)	4 (4–4)	4 (0.75–4)	4 (4–4)
(3) Timing of materials provided in advance by the research team, 0–4, median (IQR)	1.5 (0–3.25)	4 (2.25–4)	4 (3–4)	4 (4–4)	3 (3.75–4)	3 (2–3)	1.5 (0–3.25)	4 (3.75–4)	3.5 (0–4)	4 (3–4)
(4) Frequency of use of technical terms and English in explanations, 0–2, median (IQR)	2 (1.5–2)	2 (2–2)	2 (2–2)	2 (2–2)	2 (2–2)	2 (2–2)	2 (1.5–2)	2 (2–2)	2 (2–2)	2 (2–2)
(5) Difficulty of the agenda, 0–3, median (IQR)	3 (3–3)	3 (3–3)	3 (3–3)	3 (3–3)	3 (3–3)	3 (3–3)	3 (3–3)	3 (3–3)	3 (3–3)	3 (3–3)
(6) Atmosphere for the PPI contributors to speak, 0–2, median (IQR)	2 (2–2)	2 (2–2)	2 (2–2)	2 (2–2)	2 (2–2)	2 (2–2)	2 (2–2)	2 (2–2)	2 (2–2)	2 (2–2)
(7) Clarity of opinions and roles that the research team is seeking from PPI contributors, 0–2, median (IQR)	2 (2–2)	2 (2–2)	2 (2–2)	2 (2–2)	2 (2–2)	2 (2–2)	2 (2–2)	2 (2–2)	2 (2–2)	2 (2–2)
(8) Opportunities for PPI contributors to speak, 0–2, median (IQR)	2 (2–2)	2 (1.5–2)	2 (2–2)	2 (2–2)	2 (2–2)	2 (2–2)	2 (2–2)	2 (1–2)	2 (2–2)	2 (2–2)
(9) Were the opinions of the PPI contributors helpful? 0–2, median (IQR)	2 (2–2)	2 (2–2)	2 (2–2)	2 (2–2)	2 (2–2)	2 (2–2)	2 (2–2)	2 (2–2)	2 (2–2)	2 (2–2)
(10) Did the PPI contributors fulfilled the role the research team had asked of them? 0–2, median (IQR)	2 (1.5–2)	2 (2–2)	2 (2–2)	2 (2–2)	2 (2–2)	2 (2–2)	2 (1.5–2)	2 (2–2)	2 (2–2)	2 (2–2)

PPI, patient and public involvement; IQR, interquartile range

The questionnaire used for the interactive evaluation was created by PPI experts of the team (KFujisawa and KM) based on previous studies [35–37] and PPI evaluation formats [38, 39], which comprised 10 multiple-choice and two free-answer questions (Additional file 1: Table S5, S6). The original interactive evaluation is shown in Additional file 2: Figure S1 and S2. Specific items of the multiple-choice portion of the interactive evaluation were tailored as appropriate, and a 3–5-point Likert scale was used for evaluation (Table 2). Feedback obtained from this evaluation forms related to the meeting structure and preparatory materials were used for improvements in subsequent meetings. Every PPI meeting was planned for a 1–2-h duration. The meeting was video recorded and meeting minutes were drafted to allow for review of the meeting at a later time. Before publishing the research results, a final meeting was conducted with the PPI contributors on future data collection and effective content delivery to both professionals and lay persons.

## Results

### Characteristics of PPI contributors in AllerSearch app

Four PPI contributors, one man and three women (mean age = 53.0 years), were recruited for this study through the AllerSearch website and through SNS providers. All contributors had a personal or family history of suffering from hay fever.

### PPI implementation based on the eight STEPs of the AMED PPI Guidebook

Opinion exchange meetings were held nine times between February 2020 and December 2021. After the second opinion exchange meeting on March 2020, meetings were converted online to ensure the safety of participants and researchers during the city-wide lockdown issued by the Tokyo Metropolitan Government to control the spread of SARS-CoV-2. All meetings were planned 3–4 weeks in advance to ensure the participation of all PPI contributors. In each meeting, the discussion was focused on the following agenda: opinion exchange on the smartphone app and exploration of unmet medical needs (Fig. 3, STEP 1), research planning (Fig. 3, STEP 2), evaluation of prototype apps (Fig. 3, STEP 3), implementation of large-scale cloud-based clinical research (Fig. 3, STEP 5), and publication and dissemination of research results (Fig. 3, STEP 7). The discussion included numerous topics including the user interface of AllerSearch, the clarity of consent forms and questions, and the selection and quantity of the survey questionnaire.

From the first to the seventh meeting, three PPI contributors attended the meetings. One additional PPI contributor was recruited during the research process, and four PPI contributors attended the eighth and ninth meetings. Every PPI contributor participated in the research to completion without withdrawal.

### Changes in questionnaire items in AllerSearch after PPI meetings

A total of 93 items (76 added and 17 edited) in the AllerSearch survey item list were influenced in accordance with the discussion and suggestions (Table 3). Specifically, ethnicity, educational background, income, exercise, coffee intake, and supplement intake were added to the basic information (Additional file 1: Table S7). In the hay fever information section, items such as blood test results, food and drug allergies, and family history were added, as well as information on aspects of daily living, such as the clinic visited when pollen allergies occur and the cost of pollen allergies (Additional file 1: Table S7). In addition, JACQLQ [40] was added as a questionnaire offered to users, which enables researchers to evaluate patient QoL related to allergic conjunctival diseases and offer personalized recommendations (Additional file 1: Table S8). Consequently, the number of survey items increased from 97 to 165. In August 2020, a press release was issued regarding the software update of the iOS and the new release of the Android version.

**Table 3:** Changes in AllerSearch survey items based on PPI

	Before PPI	After PPI
Basic profile	Height, Weight, Age, Sibling composition, Contact lenses, Face wash, Sleep, Stool, Frequency of intake of yogurt, Materials in living room and bedroom, Pets, Tomato allergy	Height, Weight, Age, Sex, Ethnicity, **Sibling composition**, Education, Income, Current medical history, Mental illness, Smoking, Exercise, **Contact lenses**, Sleep, Stool, **Materials in living room and bedroom**, Pets, Frequency of intake of yogurt, natto, coffee, and supplements
Information about hay fever	Presence of hay fever, Age of onset, Worsening period, Atopic dermatitis	**Presence of hay fever**, **Age of onset**, **Worsening period**, Relationship with yellow sand and PM2.5, Departments to be treated, Drugs purchased at pharmacies and reasons for purchase, Allergy blood collection and its positive items, Atopic dermatitis, Urticaria, Bronchial asthma, Food allergy, Drug allergy, Family history, Impact on income, Cost of hay fever treatment
Preventative actions for hay fever	Mask, Eye drops, Nose drops, Oral medication, Air purifiers, Glasses/Goggles, Other Today's preventative actions against hay fever	Mask, Eye drops, Nose drops, Oral medication, Air purifiers, Glasses/Goggles, Wash clothes and hair when you get home, Gargle and wash your face when you get home, Moisturizer/skin care, Close windows, Hang your laundry indoors, Vaccine rice for pollen allergy, Supplements, Other Preventative behaviors of interest Duration of preventative actions against hay fever **Today’s preventative actions against hay fever**
Nasal symptom score	Runny nose, Nasal congestion, Itchy nose, Sneezing, Difficulty in daily life owing to hay fever	Runny nose, Nasal congestion, Itchy nose, Sneezing, Difficulty in daily life owing to hay fever, Number of sneezing attacks per day, Number of times you blow your nose per day
Non-nasal symptom score	Itchy eyes, Running tears, Redness of eyes, Itchy ears and mouth	Itchy eyes, Running tears, Redness of eyes, Itchy ears and mouth, Itchy skin, Abdominal pain, Headache/head heaviness, Cough, Insomnia/drowsiness, Irritability, Sluggishness, Stuffy ears
Hay fever treatment	How to apply eye drops	Oral medication, Eye drops, Nasal drops, Pastes, Injections, Laser incineration of nasal mucosa, Sublingual immunotherapy, Others **How to apply eye drops** Today’s treatment for hay fever
Dry eye	Japanese version of Ocular Surface Disease Index	Japanese version of Ocular Surface Disease Index How often do your eyes feel dry? How often do your eyes feel irritated?
Quality of life	(-)	Japanese Allergic Conjunctival Disease Quality-of-Life Questionnaire (17 questions)
Work productivity	Japanese version of Work Productivity and Activity Impairment-Allergy Specific	Japanese version of Work Productivity and Activity Impairment-Allergy Specific
Automatic acquisition	Location, Number of steps, Temperature, Humidity, PM2.5, Pollen count, Distance traveled, Moving speed, Weather	Location, Number of steps, Temperature, Humidity, PM2.5, Pollen count, Distance traveled, Moving speed, Weather
Others	Stress, Stool	Stress, Stool

Underline indicates new questions, bold indicates additions or modifications to questions

PPI, patient and public involvement; PM2.5, fine particulate matter

### Interactive evaluation questionnaire after opinion change meetings

**Fig. 4:**
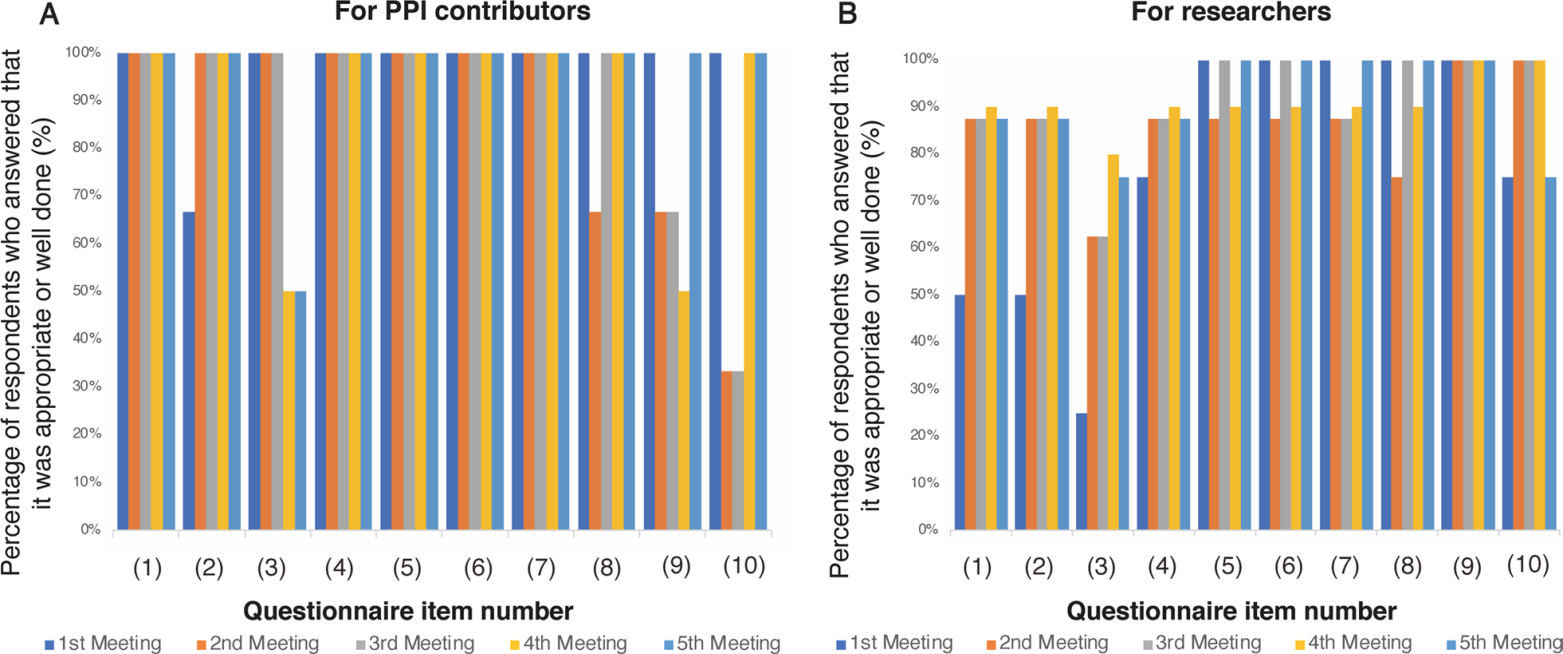
Results of the interactive evaluation. Percentage of the answers for appropriate or well done from the responses of PPI contributors (**A**) and researchers (**B**) from the first to the fifth opinion exchange meetings. PPI, patient and public involvement

Figure 4 and Table 2 shows the results of the post-meeting interactive evaluation questionnaire collected from both the PPI contributors and researchers. The level of difficulty, the atmosphere for voicing opinions, and the opportunity to speak were assessed by both researchers and PPI contributors. However, the time of receipt of relevant materials was rated low, possibly owing to the last-minute delivery of the materials and the pressure on the PPI contributors to “read” the materials before the meeting. These evaluations concern the structure and performance of each PPI meeting, such as participation opportunities and clarity regarding the PPI contributors’ roles sought by the research group. Based on the results of the survey, corrections and operational improvements were made by the following meeting.

### Building a foundation for interactive research

**Fig. 5:**
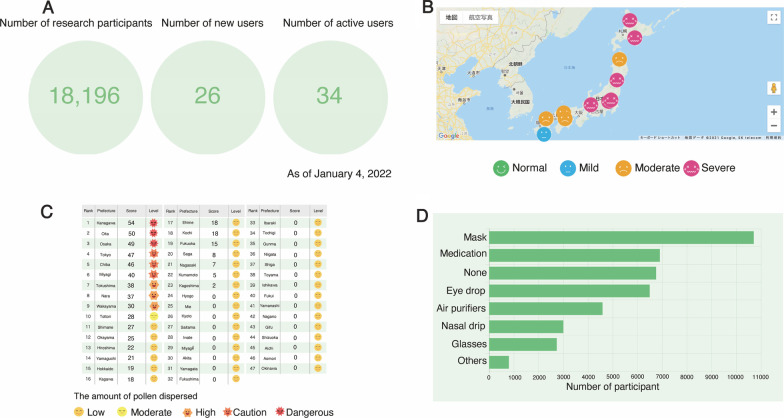
Feedback system displaying the real-time user data collected by AllerSearch on the website. **A** The number of research participants, new users, and active users. **B** Hay fever map displaying hay fever subjective symptoms of research participants expressed by a face scale based on geographical location. **C** Ranking of pollen distribution based on each prefecture. **D** Real-time user-reported preventative measures

On March 26, 2020, we developed and released a feedback system that displays the user data collected by AllerSearch on the main webpage (https://allergy-search.com/analytics/index.php). This system displays the real-time number of research participants, the number of new users, the number of active users (Fig. 5A), a hay fever map that geographically displays the overall symptom severity in a given region (Fig. 5B), hay fever severity and atmospheric pollen level table by prefecture (Fig. 5C), and a tally of user-reported preventative measures such as masks and oral medications (Fig. 5D). In addition, on August 27, 2020, an updated iOS version and a newly developed Android version of AllerSearch that incorporated the results of and suggestions from the PPI meetings were released. Notably, AllerSearch is Japan’s first smartphone app which utilizes both the ResearchKit (iOS version) and ResearchStack (Android version) platforms.

### Review of press releases

A press release was issued when the iOS version was updated, and the Android version was released on August 27, 2020. Prior to its distribution, the PPI committee contributors reviewed the press release in the opinion session. Clarity and sources of misunderstandings of the description was reviewed in the meeting.

## Discussion

Since hay fever is a multifactorial disease with a complex network of environmental, lifestyle, and host contributory risk factors, the unique perspectives and expertise offered by PPI in its study can be valuable in fulfilling previously neglected elements in performing comprehensive research. In this smartphone app-based research, we successfully incorporated various perspectives into the research process and the app by having PPI contributors take initiative as members of the research. The establishment of a structural basis for the future incorporation of PPI to mHealth app-based research, as seen through the constructive loop of communication between researchers and contributors in the development of the AllerSearch app, may have implications on promoting participatory medicine and resolving unmet medical needs.

This study implemented PPI in a systematic manner to ensure the user-friendliness of the smartphone app and to achieve stage-specific goals of research. Such methodical efforts to adopt perspectives from all angles contributes to the research quality and its comprehensiveness, aiding researchers to gain both a more expansive and deeper vision of crucial aspects on the topic of interest. The incorporation of the community encourages public awareness and insight into various conditions, contributing to a social change toward meeting unmet medical needs [9].

A smartphone app-based mHealth research has the added advantage of allowing numerous components of contributory factors to hay fever to become highly accessible in real-time with proper consent from participants [16–26]. Environmental factors using global positioning systems and correlating climate data, lifestyle factors using attached biosensors and input sources, and host factors from patient reported outcomes that include clues to individual disease activity and QoL provide expansive data on individuals’ health to form a potent database.

Notably, the barrier to app development for researchers has been reduced, owing to the release of ResearchKit (2015) and ResearchStack (2016). ResearchKit and ResearchStack are open-source frameworks aimed at developing research apps for iOS and Android devices, respectively. Since their release, the modularity of the platforms has vastly helped facilitate medical research, with more than 20 publications released using ResearchKit within the first 5 years [9]. These two frameworks are advantageous in executing large-scale observational studies aiming at analyzing real-time biometric and biosignal data with global opt-in sampling strategies.

mHealth is rapidly gaining accessibility and user-oriented qualities, showing versatility for prompt disease intervention and individually optimized management, especially for complex chronic diseases [19, 41–43]. Extensive mHealth-based measures with active user involvement in their own health holds promise for the prevention of disease onset and aggravation, enforcing the principles of P4 (predictive, preventative, personalized, and participatory) medicine [12, 21, 22, 24, 25]. The concurrent implementation of mHealth and PPI is expected to fuel the paradigm shift away from traditional hospital-oriented healthcare toward a longitudinal, person-oriented healthcare integrated into daily life.

Involving PPI contributors has been valuable and has led to improved and more meaningful research. PPI participants throughout the project made notable contributions in identifying unanticipated barriers and developing solutions. Most notably, in this study, 93 survey items were added or revised based on the results of the conducted PPI meetings (Table 3). Based on the contents of the meetings, the suggestions and proposals appear to be primarily driven by personal experiences and interactions with acquaintances and family members, which encompasses perspectives that are not always obtained through clinical conversations between providers and patients. Not only did the PPI meetings help deliver the research material to the public, but they also helped accrue invaluable perspectives and expertise from the public userbase, providing insights into various unmet medical needs. Within the study duration, an updated version of AllerSearch was released incorporating the opinions and empirical proposals collected during the PPI meetings. This underscores the compatibility of mHealth and PPI studies, as mHealth carries the advantage of being able to make instantaneous and consistent updates based on findings and suggestions gathered through PPI. In contrast, conventional PPI studies require sufficient time until formal implementation after PPI owing to the reduced flexibility of traditional research workflow. Therefore, the synergy brought by PPI implementation to an mHealth study may help bolster the quality of the research, while minimally affecting the resources and the research timeline.

The value of PPI to researchers has been highlighted [35, 44–47]. In previous reports on PPI implementation in health science research, results show that PPI helped identify complications unrecognized by researchers, gain insight on patient perspectives, and elucidate an understanding of the community of interest [35, 44–47]. This study yielded similar results, with researchers agreeing that the active exchange of opinions and post-meeting questionnaire results were insightful and helpful in improving the research quality through understanding patient viewpoints. Our results show that PPI may serve to improve the quality of research through integrating public expertise on obstacles and solutions that traditional scientists may lack, as well as establishing a trusting relationship between researchers and the target population.

While the PPI interactive evaluation form used for this study was sourced from an expert team on PPI research and referred previous studies [35–37] and PPI evaluation formats [38, 39], its validity and reliability has not yet been confirmed. Globally, publications on the impacts of PPI on research quality have nearly tripled, increasing at an unprecedented rate [13, 48]. Reports also suggest that implementation of PPI may positively affect the enrollment rates of clinical trials [15, 49]. However, despite worldwide efforts in PPI evaluation, there has been limited standardization on how the PPI impact is being measured. Further discussion from the field to create a standard for PPI assessment is warranted, as well as a thorough investigation on its validity and reliability.

This study sets a foundation for mHealth-based interactive medical care by incorporating PPI into the design of a healthcare app on hay fever: AllerSearch. Conventional research methods struggle to promptly deliver revealed findings to patients and the public. However, smartphone apps and websites enable real-time suggestions tailored to the user, as well as findings from the entire user base, offering an incentive to participate. This study seeks to act as a basis for the upcoming shift toward interactive medical care and as a platform for evidence-based medicine that promptly delivers appropriate information to patients and citizens on the management of modifiable factors related to hay fever.

There are several limitations that need to be acknowledged; while continuous efforts were made to recruit additional PPI contributors for this study, the opinion exchange meetings were held with four contributors. The necessary number of PPI contributors is not specified in the AMED guidelines [31], although it calls for efforts to seek as many diverse opinions as possible. This study had four PPI contributors, which raised concerns related to bias, such as those resulting from the limited diversity in the contributors’ age, sex, and profession. Additionally, one participant was recruited after the formal recruitment process occurred in consideration of the limited representation and diversity of the PPI contributors. The latest AMED guidelines do not explicitly state any recommendations on post-facto recruitment [31], which may create discrepancies in participants’ understanding and experiences with the ongoing research. This warrants further discussion and research regarding additional recruitments and their appropriate timing for proper PPI implementation while minimizing bias.

Devising effective methods to attract applicants is a challenge left by this study. An opinion exchange meeting was held to discuss the barriers to application and recruitment of PPI contributors in March 2021, identifying areas for improvement and the direction of future recruitment. A prevailing comment was made on the lack of perceived advantages and details of PPI participation from a public perspective, presenting as barriers to recruitment. In response, preparations have been made to update the recruitment page (e.g., photos and videos of the online opinion exchange meetings, reviews from PPI contributors) and to hold public information sessions. Future directions are aimed at resolving the barriers to entry, as well as establishing a reward system that incentivizes active participation.

## Conclusions

We presented findings to create a foundation for interactive research through incorporating principles of PPI to an mHealth research on hay fever. This fundamental structure facilitates PPI to organically gather multifaceted perspectives and expertise from public participation, contributing to a culture with societal support to improve medical care by promoting interactive research. The results may fuel future shared investigation on PPI implementation to research using digital tools, which may hold implications for the future paradigm of healthcare and P4 medicine.

## Supplementary Information

**Additional file 1: Table S1**. Survey questions before PPI. **Table S2**. Subjective symptom questionnaire for daily hay fever. **Table S3**. Questionnaire for quality of life before PPI. **Table S4**. Work productivity questionnaire. **Table S5**. Interactive evaluation questionnaire to PPI contributors. **Table S6**. Interactive evaluation questionnaire to PPI researchers. **Table S7**. Survey questions after PPI. **Table S8**. Questionnaire for quality of life after PPI.

**Additional file 2: Figure S1**. Interactive evaluation from both PPI contributors. **Figure S2**. Interactive evaluation from both researchers.

## Data Availability

Not applicable.

## Abbreviations

AMED: Japan Agency for Medical Research and Development
JACQLQ: Japanese allergic conjunctival diseases Quality of Life Questionnaire
mHealth: Mobile health
P4 medicine: Predictive, Preventative, Personalized, Participatory medicine
PPI: Patient and public involvement
QoL: Quality of life
SNS: Social networking service
