# Supplementary material for: Patient and public involvement in mobile health-based research for hay fever: a qualitative study of patient and public involvement implementation process
Source: Res Involv Engagem. 2022 Sep 2;8:45. doi: 10.1186/s40900-022-00382-6 (PMC9437402; doi:10.1186/s40900-022-00382-6)
Supplement: Supplementary file 1 — Additional file 1: Table S1. Survey questions before PPI. Table S2. Subjective symptom questionnaire for daily hay fever. Table S3. Questionnaire for quality of life before PPI. Table S4. Work productivity questionnaire. Table S5. Interactive evaluation questionnaire to PPI contributors. Table S6. Interactive evaluation questionnaire to PPI researchers. Table S7. Survey questions after PPI. Table S8. Questionnaire for quality of life after PPI. [file 40900_2022_382_MOESM1_ESM.pdf]

Supplementary Data

SUPPLEMENTARY TABLES

Supplementary Table 1. Survey questions before PPI

**Questions**	**Variables**	**Details of variables**
**User characteristics**		
	Age	Integer input, years
	Sex	Choose one {“Man,” “Woman”}
	Height	Integer input, cm
	Weight	Integer input, kg
Please provide information on your siblings.<br><br>How many siblings do you have?	Sibling	Integer input, sibling count, sibling position

What is your position among your siblings (e.g., first-born, second-born)?		
**Medical history**		
Have you ever been diagnosed with hypertension?	Medicated hypertension	Choose one {“No,” “I am being treated for hypertension,” “I have untreated hypertension,” “I do not know”}
Have you ever been diagnosed with diabetes?	Diabetes (HbA1c level)	Choose one {“Yes,” “No,” “ I do not know”}. If “Yes,” scale bar input of HbA1c level (5–15%)
Have you experienced any of the following illnesses?	Systemic diseases	Multiple choice among {“Heart disease,” “Respiratory disease,” “Brain disease,” “Liver disease,” “Kidney disease,” “Blood disease,” “Malignant tumor,” “Collagen disease,” “N/A”}

If you have eczema (atopic dermatitis), when did it start?	Atopic dermatitis	Choose one {“Infancy (before 1 year of age),” “Early childhood (age 1–6 years),” “Middle childhood (age 7–12 years),” Early adolescence (after the age of 13 years)”}
Do you experience swollen lips after consuming products containing tomato?	Tomato allergy	Choose one {“Yes,” “No”}
Do you have any mental illness?	Mental illness	Choose one {“No,” “Yes,” “Previously had”}
Do you have any of the following mental illnesses?	Mental illness	If “Yes” in the previous question, multiple choice among {“Depression,” “Schizophrenia,” “Other mental illness”}
Have you ever been diagnosed with dry eye disease?	Dry eye disease	Choose one {“No,” “Yes,” “I do not know”}

**Residential environment**		
What type of flooring do you have for your living room?	Living	Choose one {“Hardwood,” “Carpet,” “Tatami (Japanese straw-based floor),” “Vinyl,” “Other”}
What type of flooring do you have for your bedroom?	Bedroom	Choose one {“Hardwood,” “Carpet,” “Tatami (Japanese straw-based floor),” “Vinyl,” “Other”}
Do you currently own any pets?	Pet	Multiple choice among {“No,” Dog,” “Cat,” “Rabbit,” “Rodents (such as hamsters, guinea pigs),” “Birds,” “Other”}
**Lifestyle**		
How many cups of coffee do you drink per day on average?	Coffee intake	Integer input, cups

Have you ever used contact lenses?	Contact lens use	Choose one {“I have been using contact lenses,” “I have, but they were discontinued during hay fever season,” “I have used contact lenses in the past,” “I have never used contact lenses”}
What type of contact lenses do (or have) you use(d)?	Types of contact lenses	Choose one {“Soft/Daily disposable,” “Soft/Bi-weekly disposable,” “Soft/Monthly disposable,” “Soft/Yearly disposable,” “Hard,” “Colored/Daily disposable,” “Colored/Bi-weekly disposable,” “Colored/Monthly disposable,” “Colored/Yearly disposable”}
Please enter your exercise frequency and duration in the past week.	Exercise	Integer input, days, and hours

Please select the major exercise type that you undertake.	Exercise category	Choose one {“Walking,” “Light exercise,” “Bowling” “Swimming,” “Equipment-based training,” “Jogging,” “Marathon,” “Hiking,” “Cycling,” “Fishing,” “Golfing (including indoor ranges),” “Baseball (including catch ball),” “Other”}
Are you using eye drops (artificial tears)?	Eye drops	Choose one {“Currently using (non-contact lens user),” “Currently using without removing contact lens (contact lens user),” “Currently using after removing contact lenses (contact lens user),” “Not currently using”}

Do you use eye wash solutions? If yes, at what time of the day do you use them?	Eye wash	Multiple choice among {"Morning," "Afternoon," "Evening," "Before sleeping," "When symptoms worsen," "Do not use eye wash"}
Please enter the average frequency of bowel movements per week.	Bowel movements	Integer input, times
Please enter your average sleep duration per day.	Sleep duration	Integer input, hours
Do you currently smoke or have you smoked in the past?	Smoking	Choose one {"No," "Yes," "Have before"} If "Yes" or "Have before," scale bar input of Number of cigarettes per day, Number of years smoked
How many times do you consume yogurt (or yogurt-containing products) per week?	Yogurt intake	Choose one {"Rarely," "Once a week," "Twice or thrice a week," "Four or five times a week," "Everyday"}

**Hay fever**		
Do you have hay fever?	Hay fever	Choose one {“Yes,” “No,” “Unknown”}
When did your hay fever start?	Hay fever onset	Integer input, years old
In which months of the year does your hay fever occur?	Months when hay fever develops	Multiple choice among {“January,” “February,” “March,” “April,” “May,” “June,” “July,” “August,” “September,” “October,” “November,” “December”}
In which months of the year does your hay fever worsen?	Months when hay fever worsens	Multiple choice among {“January,” “February,” “March,” “April,” “May,” “June,” “July,” “August,” “September,” “October,” “November,” “December”}

Have you ever received subcutaneous or sublingual allergen immunotherapy (desensitization/hyposensitization)?	Sublingual and subcutaneous desensitization therapy	Choose one {“Yes,” “No”}
Please tell us which of the following hay fever prevention methods you have been using.	Preventive behavior	Multiple choice among {“Mask,” “Eye drops,” “Nasal spray/drops,” “Medication,” “Air purifier,” “Glasses and goggles,” “Other,” “Not using any”}
**Dry eye questionnaire**		
Have you experienced any of the following during the last week? Eyes that are sensitive to light?	J-OSDI item 1	Choose one {“None of the time, “Some of the time,” “Half of the time, “Most of the time,” “ All of the time”}
Have you experienced any of the following during the last week? Eyes that are feel gritty?	J-OSDI item 2	

Have you experienced any of the following during the last week? Painful or sore eyes?	J-OSDI item 3	
Have you experienced any of the following during the last week? Blurred vision?	J-OSDI item 4	
Have you experienced any of the following during the last week? Poor vision?	J-OSDI item 5	
Have problems with your eyes limited you preforming any of the following during the last week? Reading?	J-OSDI item 6	
Have problems with your eyes limited you preforming any of the following during the last week? Driving at night?	J-OSDI item 7	

Have problems with your eyes limited you preforming any of the following during the last week? Working with a computer or bank machine (ATM)?	J-OSDI item 8	
Have problems with your eyes limited you preforming any of the following during the last week? Watching TV?	J-OSDI item 9	
Have your eyes felt uncomfortable in any of the following situations during the last week? Windy conditions?	J-OSDI item 10	

Have your eyes felt uncomfortable in any of the following situations during the last week? Places or areas with low humidity (very dry)?	J-OSDI item 11	
Have your eyes felt uncomfortable in any of the following situations during the last week? Areas that are air conditioned?	J-OSDI item 12	

Abbreviations: PPI, patient and public involvement; HbA1c, glycated hemoglobin; N/A, not applicable; J-OSDI, Japanese version of Ocular Surface Disease Index.

Supplementary Table 2. Subjective symptom questionnaire for daily hay fever

Questions	Variables	Details of variables
**Nasal symptoms score**		
Please rate how your rhinorrhea has been over the past 24 hours.	NSS item 1	Choose one {“No symptoms,” “Mild symptoms (symptoms clearly present but easily tolerated,” “Moderate symptoms (symptoms bothersome but tolerable),” “Severe symptoms (symptoms difficult to tolerate—interfere with activities)”}
Please rate how your nasal congestion has been over the past 24 hours.	NSS item 2	
Please rate how your nasal itching has been over the past 24 hours.	NSS item 3	

Please rate how your sneezing has been over the past 24 hours.	NSS item 4	
How severely does hay fever affect your daily activities?	NSS item 5	
How many sneezing attacks do you have per day?	NSS item 6	Choose one {"None," "1–5 times," "6–10 times," "11–20 times, "More than 21 times"}
How many times do you blow your nose per day?	NSS item 7	
How often do you breathe through your mouth owing to nasal congestion?	NSS item 8	Choose one {"None," "No mouth breathing but some nasal congestion, "Sometimes," "Most of the time, "All day"}
**Non-nasal symptoms score**		

Please rate how your eye itching has been over the past 24 hours.	NNSS item 1	Choose one {“No symptoms,” “Mild symptoms (symptoms clearly present but easily tolerated,” “Moderate symptoms (symptoms bothersome but tolerable),” “Severe symptoms (symptoms difficult to tolerate—interfere with activities)”}
Please rate how your eye-watering has been over the past 24 hours.	NNSS item 2	
Please rate how your eye redness has been over the past 24 hours.	NNSS item 3	
Please rate how your itching of the ear and nose has been over the past 24 hours.	NNSS item 4	

Please rate how your itching of skin has been over the past 24 hours.	NNSS item 5a	
Have you experienced any of the following symptoms over the past 24 hours?	NNSS item 5b	Multiple choice among {“No,” “Itchy skin,” “Abdominal pain,” “Headache/head heaviness,” “Cough,” “Insomnia/drowsiness,” “Irritability,” “Sluggishness,” “Stuffy ears”}
Please tell your stress level on a scale of one to five	Stress level	Slider input, 0–5
Please tell us how many steps you took yesterday.	Number of steps	Choose one {“0–999 steps,” “1000–1999 steps,” “2000–2999 steps,” 3000–3999 steps, “4000–4999 steps,” “5000–5999 steps,” “6000–6999 steps,” “7000–7999 steps,” “8000–8999 steps,” “9000–9999 steps,” “10 000–10 999

		steps," "11 000–11 999 steps," "12 000–12 999 steps," "13 000–13 999 steps," "14 000–14 999 steps, "More than 15 000 steps"}
Please tell us about the nature of yesterday's stool.	Stool	Choose one {"Hard, "Normal," "Soft," "Muddy," "Watery," "Bloody," "Sour," "Smell," "No stool yesterday"}
What is your preventive behavior for hay fever today?	Preventive behavior	Multiple choice among {"Mask," "Eye drops," "Nasal spray/drops," "Medication," "Air purifier," "Glasses and goggles," "Wash clothes and hair," "Gargle and wash eyes," "Moisturize and skin care," "Close windows," "Avoid drying futons and laundry outside," "Pollen rice, "Supplements," "Other," "Not using any"}
What is today's treatment for hay fever?	Treatment	Multiple choice among {"Internal medicine," "Eye drops," "Nasal spray/drops," "Pastes," "Injections," "Nasal mucosal laser ablation," "Sublingual immunotherapy," "Other," "Not applicable"}

Taking photo of the lower eyelid conjunctiva	Photo	

Abbreviations: NSS, nasal symptom score; NNSS, non-nasal symptom score

Supplementary Table 3. Questionnaire for quality of life before PPI

Questions	Variables	Details of variables
**Quality of life questionnaire**		
Please check the degree to which the following quality of life questions were most severe in the last 1–2 weeks owing to nasal/eye symptoms. Please check "none" for items that are clearly not related to nasal/ocular symptoms.		
Obstacles to studying, working, and housework	QoL item 1	Choose one {"None, "Mild," "Soft," " Somewhat severe" " Severe," " Very severe"}
Poor mental concentration	QoL item 2	
Decreased thinking ability	QoL item 3	

Impaired reading newspapers and other materials	QoL item 4	
Poor memory	QoL item 5	
Limitation of outdoor life such as sports and picnics	QoL item 6	
Limitation of going out	QoL item 7	
Obstacles to socializing with people	QoL item 8	
Interfering with conversations and telephone calls with others	QoL item 9	
Anxiety about people around you	QoL item 10	

Sleeping disorder	QoL item 11	
Dullness	QoL item 12	
Fatigue	QoL item 13	
Frustrated	QoL item 14	
Irritable	QoL item 15	
Depressed	QoL item 16	
Dissatisfaction with life	QoL item 17	
Please check the number on the face that describes your general condition (including symptoms, life, and feelings) in the last 1–2 weeks.	QoL item 18	Choose one from face scale

Abbreviations: PPI, patient and public involvement; QoL, quality of life

Supplementary Table 4. Work productivity questionnaire

**Questions**	**Variables**	**Details of variables**
Are you currently employed? (Do you have a remunerative job?)	Work	Choose one {"No," "Yes"}
How many hours did you actually work in the past 7 days?	Work hours	Integer input, hours
How many hours did you miss work owing to hay fever in the past 7 days?	Impact of hay fever on working	Integer input, hours

During the past 7 days at work, how much did hay fever affect your productivity (the amount and type of work that could be done)?	Impact of hay fever on work productivity	Choose one {0–10}
Are you currently a student?	Student	Choose one {“No,” “Yes”}
How many hours of class did you take in the past 7 days?	Study hours	Integer input, hours
How many hours of class did you miss in the past 7 days owing to hay fever?	Impact of hay fever on studying	Integer input, hours

How much did your hay fever affect your studies during the past 7 days of class?	Impact of hay fever on productivity	Choose one {0–10}
How much did hay fever affect your daily activities (except work and school activities) in the past 7 days	Impact of hay fever on productivity2	Choose one {0–10}

Supplementary Table 5. Interactive evaluation questionnaire to PPI contributors

**Questions**	**Details of variables**
(1) Difficulty of the materials provided in advance by the research team	Choose one {“Appropriate level of difficulty, “Difficult,” “Easy,” “Not provided “Not involved so don’t know”}
(2) Number of materials provided in advance by the research team	Choose one {“Appropriate amount, “Too much,” “Too little,” “Not provided “Not involved so don’t know”}
(3) Timing of materials provided in advance by the research team	Choose one {“Appropriate, “Late,” “Early,” “Not provided “Not involved so don’t know”}
(4) Frequency of use of technical terms and English in explanations	Choose one {“Appropriately used, “Frequently used, “Undecided”}
(5) Difficulty of the agenda	Choose one {“Appropriately level of difficulty, “Difficult,” “Easy,” “Undecided”}

(6) Atmosphere for the PPI contributors to speak	Choose one {“Easy to speak up, “Difficult to speak up,” “Can’t say either”}
(7) Clarity of opinions and roles that the research team is seeking from PPI contributors	Choose one {“Clear, “Unclear,” “Undecided”}
(8) Opportunities for PPI contributors to speak	Choose one {“Provided appropriately, “Asked to speak too many times,” “More opportunities to speak should have been provided”}
(9) Were you able to say what you wanted to say?	Choose one {“Yes, “No,” “Can’t say either”}
(10) Did the PPI contributors fulfilled the role the research team had asked of them?	Choose one {“Yes, “No,” “Can’t say either”}
(11) If there is anything you learned from PPI contributors in today’s opinion exchange meeting, please specify	Free writing

(12) Please indicate what you think should be continued or improved about future opinion exchange meetings	Free writing

Abbreviations: PPI, patient and public involvement

Supplementary Table 6. Interactive evaluation questionnaire to PPI researchers

**Questions**	**Details of variables**
(1) Difficulty of the materials provided in advance by the research team	Choose one {"Appropriate level of difficulty, "Difficult," "Easy," "Not provided "Not involved so don't know"}
(2) Number of materials provided in advance by the research team	Choose one {"Appropriate amount, "Too much," "Too little," "Not provided "Not involved so don't know"}
(3) Timing of materials provided in advance by the research team	Choose one {"Appropriate, "Late," "Early," "Not provided," "Not involved so don't know"}
(4) Frequency of use of technical terms and English in explanations	Choose one {"Appropriately used, "Frequently used, "Undecided"}
(5) Difficulty of the agenda	Choose one {"Appropriately level of difficulty, "Difficult," "Easy," "Undecided"}

(6) Atmosphere for the PPI contributors to speak	Choose one {“Easy to speak up, “Difficult to speak up,” “Can’t say either”}
(7) Clarity of opinions and roles that the research team is seeking from PPI contributors	Choose one {“Clear, “Unclear,” “Undecided”}
(8) Opportunities for PPI contributors to speak	Choose one {“Provided appropriately,” “Asked to speak too many times,” “More opportunities to speak should have been provided”}
(9) Were the opinions of the PPI contributors helpful?	Choose one {“Yes, “No,” “Can’t say either”}
(10) Did the PPI contributors fulfilled the role the research team had asked of them?	Choose one {“Yes,” “No,” “Can’t say either”}
(11) If there is anything you learned from PPI contributors in today’s opinion exchange meeting, please specify	Free writing

(12) Please indicate what you think should be continued or improved about future opinion exchange meetings	Free writing

Abbreviations: PPI, patient and public involvement

Supplementary Table 7. Survey questions after PPI

**Questions**	**Variables**	**Details of variables**
**User characteristics**		
	Age	Integer input, years
	Sex	Choose one {"Man," "Woman"}
	Height	Integer input, cm
	Weight	Integer input, kg
	Ethnicity	Choose one {"Japanese," "The others"}
Please provide information on your siblings.	Sibling	Integer input, sibling position

What is your position among your siblings (e.g., first-born, second-born)?		
Are you currently enrolled in a school?	Education	Choose one {“No,” “Yes”}
Please provide information about your last educational background.	Last educational background	Choose one {“Elementary or middle school,” “High School, “Vocational school,” “Junior college,” “University, “Graduate school”}
Please provide information about your annual income (Individual income, not household income).	Income	Choose one {“0–1.99 million yen,” “2–3.99 million yen,” “4–5.99 million yen,” “6–7.99 million yen,” “8–9.99 million yen,” “10 million yen,” “15 million yen,” “20 million yen”}

**Medical history**		
Have you experienced any of the following illnesses? (Excluding mental illness)	Medical history	Multiple choice among {“Allergic disease,” “Hypertension,” “Diabetes,” “Hyperlipidemia,” “Heart disease,” “Respiratory disease,” “Brain disease,” “Liver disease,” “Kidney disease,” “Thyroid disease,” “Blood disease,” “Malignant tumor,” “Collagen disease,” “Glaucoma,” Other,” “N/A”}
Have you ever been diagnosed with mental illness?	Mental illness	Multiple choice among {“Depression,” “Anxiety,” “Schizophrenia,” “Manic depression,” “Other mental illness,” “N/A”}
**Hay fever**		

Do you have hay fever?	Hay fever	Choose one {“No,” “Currently have,” “Previously had but healed spontaneously,” “Previously had but currently treated and cured,” “I do not know”}
When did your hay fever start?	Hay fever onset	Integer input, years old
In which months of the year does your hay fever worsen?	Months when hay fever develops	Multiple choice among {“January,” “February,” “March,” “April,” “May,” “June,” “July,” “August,” “September,” “October,” “November,” “December”}
Are hay fever symptoms worse when yellow sand or PM2.5 is flying around?	Yellow sand and PM2.5	Choose one {“No,” “Yes,” “I do not know”}

Which medical department do you visit when you have hay fever?	Medical department	Multiple choice among {“I do not visit,” “ENT,” “Ophthalmology,” “Dermatology,” “Allergy,” “Internal Medicine,” “Pediatrics,” “Drug store,” “Other department”}
What kind of drugs do you buy at the drug store?	Drug store	If “Drug store” in the previous question, multiple choice among {“Eye drops,” “Nasal drops,” “Oral medicine,” “Ointment,” “Supplements,” “Other”}
Why did you buy the medicine at the drug store?	Drug store	If “Drug store” in the previous question, multiple choice among {“Inexpensive,” “Time-consuming,” “Convenient,” “ Over-the-counter medicine is enough,” “I can choose my own medicine,” “I can consult with a pharmacist,” “I want to prevent,” “Other”}
**Allergy**		

Have you ever had blood test for allergens?	Blood test for allergens	Choose one {"No," "Yes"}
Which of the following items in the blood test are positive?	Allergens that are positive	If "Yes" in the previous question, multiple choice among {"Japanese cedar," "Japanese cypress," "Japanese birch/alder," "Rice," "Ragweed," "Mug wort," "Japanese anemone," " Mite/house dust," "Other," "No allergy positive items," "I do not know"}
If you have eczema (atopic dermatitis), when did it start?	Atopic dermatitis	Choose one {"Infancy (before 1 year of age)," "Early childhood (age 1–6 years)," "Middle childhood (age 7–12 years)," Early adolescence (after the age of 13 years)"}

If you have urticaria, when did it start?	Urticaria	Choose one {“Infancy (before 1 year of age),” “Early childhood (age 1–6 years),” “Middle childhood (age 7–12 years),” Early adolescence (after the age of 13 years)”}
If you have asthma, when did it start?	Asthma	Choose one {“Infancy (before 1 year of age),” “Early childhood (age 1–6 years),” “Middle childhood (age 7–12 years),” Early adolescence (after the age of 13 years)”}
If you have food allergy, when did it start?	Food allergy	Choose one {“Infancy (before 1 year of age),” “Early childhood (age 1–6 years),” “Middle childhood (age 7–12 years),” Early adolescence (after the age of 13 years)”}
Have you ever had a food allergy to any of the following foods?	Food allergy	Multiple choice among {“Tomato,” “Watermelon,” “Melon,” “Kiwi,” “Apple,” “Cherry,” “Potato,” “ Peach,” “Other”}

If you have drug allergy, when did it start?	Drug allergy	Choose one {"Infancy (before 1 year of age)," "Early childhood (age 1–6 years)," "Middle childhood (age 7–12 years)," Early adolescence (after the age of 13 years)"}
Is there anyone in your family who has hay fever?	Family history of hay fever	Multiple choice among {"No," "Father," "Mother," "Brother/Sister," "Child"}
**Preventive behavior for hay fever**		
Please provide information about which of the following hay fever prevention methods you have been using.	Preventive behavior	Multiple choice among {"Mask," "Eye drops," "Nasal spray/drops," "Medication," "Air purifier," "Glasses and goggles," "Wash clothes and hair," "Gargle and wash eyes," "Moisturize and skin care," "Close windows," "Avoid drying futons and laundry outside," "Pollen rice, "Supplements," "Other," "Not using any"}

What kind of hay fever treatment do you take?	Hay fever treatment	Multiple choice among {"Internal medicine," "Eye drops," "Nasal spray/drops," "Pastes," "Injections," "Nasal mucosal laser ablation," "Sublingual immunotherapy," "Other," "Not applicable"}
When do you usually take preventive behaviors against hay fever?	The month to start taking preventive behaviors against hay fever	Choose one {"January," "February," "March," "April," "May," "June," "July," "August," "September," "October," "November," "December," I don't take preventive behaviors"}
Please tell us about the hay fever countermeasures you are interested in.	Countermeasures	Multiple choice among {"Mask," "Eye drops," "Nasal spray/drops," "Medication," "Air purifier," "Glasses and goggles," "Wash clothes and hair," "Gargle and wash eyes," "Moisturize and skin care," "Close windows," "Avoid drying

		futons and laundry outside," "Pollen rice, "Supplements," "Other," "Not interested any"}
What percentage of your income do you think you will lose financially by suffering from hay fever?	Economic loss	Slider input, 0–100%
How much per month do you think you can spend on hay fever control?	Cost	Choose one {"0–999 yen," "1000–2999 yen," "3000–4999 yen," "5000–7999 yen," "8000–9999 yen," "10 000–19 999 yen," "more than 20 000 yen"}
**Dry eye**		

Have you ever been diagnosed (by a clinician) as having dry eye syndrome?	Diagnosis	Choose one {"No," "Yes," "I do not know"}
How often do your eyes feel dry (not wet enough)?	Frequency of feeing dry	Choose one {"Never," "Sometimes," "Often," "Constantly"}
How often do your eyes feel irritated?	Frequency of feeing irritated	Choose one {"Never," "Sometimes," "Often," "Constantly"}
Have you experienced any of the following during the last week? Eyes that are sensitive to light?	J-OSDI item 1	Choose one {"None of the time, "Some of the time," "Half of the time, "Most of the time," " All of the time"}

Have you experienced any of the following during the last week? Eyes that are feel gritty?	J-OSDI item 2	
Have you experienced any of the following during the last week? Painful or sore eyes?	J-OSDI item 3	
Have you experienced any of the following during the last week? Blurred vision?	J-OSDI item 4	
Have you experienced any of the following during the last week? Poor vision?	J-OSDI item 5	

Have problems with your eyes limited you preforming any of the following during the last week? Reading?	J-OSDI item 6	
Have problems with your eyes limited you preforming any of the following during the last week? Driving at night?	J-OSDI item 7	
Have problems with your eyes limited you preforming any of the following during the last week?	J-OSDI item 8	

Working with a computer or bank machine (ATM)?		
Have problems with your eyes limited you preforming any of the following during the last week? Watching TV?	J-OSDI item 9	
Have your eyes felt uncomfortable in any of the following situations during the last week? Windy conditions?	J-OSDI item 10	
Have your eyes felt uncomfortable in any of the following situations	J-OSDI item 11	

during the last week? Places or areas with low humidity (very dry)?		
Have your eyes felt uncomfortable in any of the following situations during the last week? Areas that are air conditioned?	J-OSDI item 12	
**Residential environment**		
What type of flooring do you have for your living room?	Living	Choose one {"Hardwood," "Carpet," "Tatami (Japanese straw-based floor)," "Other"}
What type of flooring do you have for your bedroom?	Bedroom	Choose one {"Hardwood," "Carpet," "Tatami (Japanese straw-based floor)," "Other"}

Do you currently own any pets?	Pet	Multiple choice among {“No,” Dog,” “Cat,” “Rabbit,” “Rodents (such as hamsters, guinea pigs),” “Birds,” “Other”}
**Lifestyle**		
Do you currently smoke or have you smoked in the past?	Smoking	Choose one {“No,” “Yes,” “Have before”} If “Yes” or “Have before,” scale bar input of Number of cigarettes per day, Number of years smoked
How long have you smoked cigarettes?	Years of smoking	If “Yes” or “Have before,” Integer input, years of smoking
Please provide information on how much time you’ve spent outdoors in the past week.	Outdoor Activities	Integer input, hours

Have you ever used contact lenses?	Contact lens use	Choose one {“I have been using contact lenses,” “I have, but they were discontinued during hay fever season,” “I have used contact lenses in the past,” “I have never used contact lenses”}
What type of contact lenses do (or have) you use(d)?	Types of contact lenses	If not “I have never used contact lenses” in the previous question, choose one {“ Soft/Daily disposable,” “ Soft/Bi-weekly disposable,” “ Soft/Monthly disposable,” “ Soft/Yearly disposable,” “Hard,” “Orthokeratology”}
Are you using eye drops for hay fever symptoms?	Eye drops	Choose one {“Currently using (non-contact lens user),” “Currently using without removing contact lens (contact lens user),” “Currently using without removing contact lens (contact lens user),” “Not currently using”}

Please enter your average sleep duration per day.	Sleep duration	Integer input, hours
Please enter the average frequency of bowel movements per week.	Bowel movements	Integer input, times
How many times do you consume natto (fermented soybeans) per week?	Natto intake	Choose one {“Rarely,” “Once a week,” “Twice or thrice a week,” “Four or five times a week,” “Everyday”}
How many times do you consume yogurt (or yogurt-containing products) per week?	Yogurt intake	Choose one {“Rarely,” “Once a week,” “Twice or thrice a week,” “Four or five times a week,” “Everyday”}
Do you take any supplements?	Supplement	Choose one {“No,” “Yes”}

How many cups of coffee do you drink in a day?	Coffee intake	Integer input, cups

Abbreviations: PPI, patient and public involvement; N/A, not applicable; PM2.5, fine particulate matter; J-OSDI, Japanese version of Ocular Surface Disease Index.

Supplementary Table 8. Questionnaire for quality of life after PPI

Questions	Variables	Details of variables
**Japanese allergic conjunctival disease quality of life questionnaire**		
Have you had any of the following nasal or eye symptoms in the last 1–2 weeks? Please choose the number that best describes the severity.		
Itchy eyes	JACQLQ item 1	Choose one {“No symptoms,” “Mild symptoms,” “Moderate symptoms,” “Severe symptoms,” “Very severe symptoms”}
Rumbling of eyes	JACQLQ item 2	

Red eyes	JACQLQ item 3	
Teary eyes	JACQLQ item 4	
Eye discharge	JACQLQ item 5	
Runny nose	JACQLQ item 6	
Sneeze	JACQLQ item 7	
Frequency of mouth breathing owing to nasal congestion	JACQLQ item 8	

Itchy nose	JACQLQ item 9	
**Quality of life questionnaire**		
Please check the degree to which the following quality of life questions were most severe in the last 1–2 weeks owing to nasal/ocular symptoms. Please check "none" for items that are clearly not related to nasal/ocular symptoms.		
About interference with study, work	JACQLQ item 10	Choose one {“None,” “Mild,” “Soft,” “ Somewhat severe” “ Severe,” “Very severe”}

About poor mental concentration	JACQLQ item 11	
About decreased ability to think (inability to think clearly).	JACQLQ item 12	
About interference with newspapers and reading (inconvenience)	JACQLQ item 13	
About memory loss (poor recall)	JACQLQ item 14	
About hindrance to outdoor activities such as sports, picnics	JACQLQ item 15	

About hindrance to going out (tend to refrain from going out)	JACQLQ item 16	
About hindrance to socializing (tend to refrain from socializing)	JACQLQ item 17	
About Interference with conversations or phone calls with others	JACQLQ item 18	
I care about the people around me.	JACQLQ item 19	
About sleep disorders (not sleeping well)	JACQLQ item 20	

About fatigue (tiredness)	JACQLQ item 21	
About tiredness (easy to get tired)	JACQLQ item 22	
About feeling unwell	JACQLQ item 23	
About irritability	JACQLQ item 24	
About depression	JACQLQ item 25	
About dissatisfaction with life	JACQLQ item 26	

Please check the number on the face that describes your general condition (including symptoms, life, and feelings) in the last 1–2 weeks.	JACQLQ item 27	Choose one from face scale

Abbreviations: PPI, patient and public involvement; JACQLQ, Japanese allergic conjunctival disease quality of life questionnaire; QoL, quality of life
