# Supplementary material for: Patient and public involvement in mobile health-based research for hay fever: a qualitative study of patient and public involvement implementation process
Source: Res Involv Engagem. 2022 Sep 2;8:45. doi: 10.1186/s40900-022-00382-6 (PMC9437402; doi:10.1186/s40900-022-00382-6)
Supplement: Supplementary file 2 — Additional file 2: Figure S1. Interactive evaluation from both PPI contributors. Figure S2. Interactive evaluation from both researchers. [file 40900_2022_382_MOESM2_ESM.pdf]

Supplementary Data

SUPPLEMENTARY FIGURE

Supplementary Figure 1. Interactive evaluation from both PPI contributors.

For PPI contributors

Thank you very much for your participation today. We would like to hear your opinions and impressions so that we can improve our activities for patient and public involvement (PPI) in the future.

1. Your name (   )

2. Please share your evaluation of today's opinion exchange meeting. (Check only one)

(1) Difficulty of the materials provided in advance by the research team

Appropriate level of difficulty / Difficult / Easy / Not provided / Not involved so don't know

(2) Amount of materials provided in advance by the research team

Appropriate amount / Too much / Too little / Not provided / Not involved so don't know

(3) Timing of materials provided in advance by the research team

Appropriate / Late / Early / Not provided / Not involved so don't know

(4) Frequency of use of technical terms and English in explanations

Appropriately used / Frequently used / Undecided

(5) Difficulty of the agenda

Appropriate level of difficulty / Difficult / Easy / Undecided

(6) Atmosphere for the PPI contributors to speak

Easy to speak up / Difficult to speak up / Can't say either

(7) Clarity of opinions and roles the research team is seeking from PPI contributors

Clear / Unclear / Undecided

(8) Opportunities for PPI contributors to speak

Provided appropriately / Asked to speak too many times / More opportunities to speak should have been provided

(9) Were you able to say what you wanted to say?

Yes / No / Can't say either

(10) Did the PPI contributors fulfill the role the research team had asked of them?

Yes / No / Can't say either

3. If there is anything you learned from the PPI contributors in today's opinion exchange meeting, please specify.

4. Please indicate what you think should be continued or improved in future opinion exchange meetings.

Thank you for your cooperation!

Abbreviations: PPI, patient and public involvement

Supplementary Figure 2. Interactive evaluation from both researchers.

For PPI researchers

Thank you very much for your participation today. We would like to hear your opinions and impressions so that we can improve our activities for patient and public involvement (PPI) in the future.

1. Your name (   )

2. Please share your evaluation of today's opinion exchange meeting. (Check only one)

(1) Difficulty of the materials provided in advance by the research team

Appropriate level of difficulty / Difficult / Easy / Not provided / Not involved so don't know

(2) Amount of materials provided in advance by the research team

Appropriate amount / Too much / Too little / Not provided / Not involved so don't know

(3) Timing of materials provided in advance by the research team

Appropriate / Late / Early / Not provided / Not involved so don't know

(4) Frequency of use of technical terms and English in explanations

Appropriately used / Frequently used / Undecided

(5) Difficulty of the agenda

Appropriate level of difficulty / Difficult / Easy / Undecided

(6) Atmosphere for the PPI contributors to speak

Easy to speak up / Difficult to speak up / Can't say either

(7) Clarity of opinions and roles that the research team is seeking from PPI contributors

Clear / Unclear / Undecided

(8) Opportunities for PPI contributors to speak

Provided appropriately / Asked to speak too many times / More opportunities to speak should have been provided

(9) Were the opinions of the PPI contributors helpful?

Helpful / Not helpful / Can't say either

(10) Did the PPI contributors fulfill the role the research team had asked of them?

Yes / No / Can't say either

3. If there is anything you learned from the PPI contributors in today's opinion exchange meeting, please specify.

4. Please indicate what you think should be continued or improved in future opinion exchange meetings.

Thank you for your cooperation!

Abbreviations: PPI, patient and public involvement
